# *KRAS* Mutation Status and Treatment Outcomes in Patients With Metastatic Pancreatic Adenocarcinoma

**DOI:** 10.1001/jamanetworkopen.2024.53588

**Published:** 2025-01-07

**Authors:** Carter Norton, Matthew Steven Shaw, Zachary Rubnitz, Jarrod Smith, Heloisa P. Soares, Christopher D. Nevala-Plagemann, Ignacio Garrido-Laguna, Vaia Florou

**Affiliations:** 1 Huntsman Cancer Institute, University of Utah Health Care, Salt Lake City; 2 McKusick-Nathans Institute of Genetic Medicine, Johns Hopkins University School of Medicine, Baltimore, Maryland; 3 Department of Internal Medicine, Division of Oncology, University of Utah Health Care, Salt Lake City; 4 Department of Internal Medicine, University of Utah Health Care, Salt Lake City

## Abstract

**Importance:**

Despite the high prevalence of *KRAS* alterations in pancreatic ductal adenocarcinoma (PDAC), the clinical impact of common *KRAS* mutations with different cytotoxic regimens is unknown. This evidence is important to inform current treatment and provide a benchmark for emergent targeted *KRAS* therapies in metastatic PDAC.

**Objective:**

To assess the clinical implications of common *KRAS* G12 mutations in PDAC and to compare outcomes of standard-of-care multiagent therapies across these common mutations.

**Design, Setting, and Participants:**

This retrospective cohort study obtained deidentified clinical data for 5382 patients from a nationwide (US-based) clinicogenomic database. The deidentified data originated from approximately 280 US cancer clinics (approximately 800 sites of care). Patients diagnosed with metastatic PDAC from February 9, 2010, to September 20, 2022, and with sufficient follow-up and treatment data were included.

**Main Outcomes and Measures:**

Median overall survival (OS) and time to next treatment (TTNT) were calculated for each *KRAS* mutation group. Hazard ratios (HRs) were generated using multivariate Cox proportional hazards models for *KRAS* mutations and mutation-treatment combinations.

**Results:**

A total of 2433 patients with PDAC were included in the analysis (mean age at first treatment, 67.0 [range, 66.0-68.0] years; 1340 male [55.1%]). Among 2023 patients with *KRAS* mutations, those with G12R had the longest median TTNT (6.0 [95% CI, 5.2-6.6] months) and the longest median OS (13.2 [95% CI, 10.6-15.2] months). Patients with *KRAS* G12D and G12V mutations had a significantly higher risk of disease progression (HRs, 1.15; [95% CI, 1.04-1.29; *P* = .009] and 1.16 [95% CI, 1.04-1.30; *P* = .01], respectively) and death (HRs, 1.29 [95% CI, 1.15-1.45; *P* < .001] and 1.23 [95% CI, 1.09-1.39; *P* < .001], respectively) compared with *KRAS* wild type. The FOLFIRINOX regimen (fluorouracil, irinotecan, oxaliplatin, and leucovorin) had a significantly lower risk of treatment progression and death than gemcitabine with (HRs, 1.19 [95% CI, [1.09-1.29; *P* < .001] and 1.18 [95% CI, 1.07-1.29; *P* < .001], respectively) or without (HRs, 1.37 [95% CI, 1.11-1.69; *P* = .003] and 1.41 [95% CI 1.13-1.75; *P* = .002], respectively) nab-paclitaxel across all patients.

**Conclusions and Relevance:**

In this cohort study of 2433 patients with PDAC, *KRAS* G12D and G12V mutations were associated with worse patient outcomes compared with *KRAS* wild type. *KRAS* G12R was associated with more favorable patient outcomes, and FOLFIRINOX was associated with better patient outcomes than gemcitabine-based therapies. These findings highlight the need for more effective systemic therapies for these groups of patients.

## Key Points

**Question:**

Are specific *KRAS* mutations associated with different first-line treatment outcomes in metastatic pancreatic ductal adenocarcinoma?

**Findings:**

In this cohort study of 2433 patients, those with *KRAS* G12D or G12V mutations had a significantly higher risk of disease progression (hazard ratios, 1.15 and 1.16, respectively) and death (hazard ratios, 1.29 and 1.23, respectively) compared with *KRAS* wild type. In contrast, *KRAS* G12R mutations were associated with better outcomes, and FOLFIRINOX treatment was associated with better outcomes compared with gemcitabine-based regimens.

**Meaning:**

The differential association of *KRAS* mutations in metastatic pancreatic ductal adenocarcinoma highlights the need for more effective systemic therapies for these patients.

## Teaser

This cohort study assesses the incidence of *KRAS* mutations and their association with clinical outcomes based on the most common treatments used in patients with pancreatic ductal adenocarcinoma.

## Introduction

Pancreatic ductal adenocarcinoma (PDAC) is predicted to become the second leading cause of cancer-related deaths by 2040,^1^ and most patients present with incurable disease. Treatment for pancreatic cancer in localized and metastatic settings has made only modest progress in recent years.^2,3^ The 5-year survival rate (12%) remains one of the lowest among patients with cancer.^4^ In patients with metastatic disease, first-line regimens include modified FOLFIRINOX (fluorouracil, irinotecan, oxaliplatin, and leucovorin) and nab-paclitaxel plus gemcitabine based on results from 2 trials: PRODIGE (Partenariat de Recherche en Oncologie Digestive) and MPACT (Matching Patients to Accelerate Clinical Trials).^5,6^ In the PRODIGE trial, the median survival with FOLFIRINOX compared with gemcitabine was 11.1 vs 6.8 months (hazard ratio [HR], 0.57; 95% CI, 0.45-0.73; *P* < .001).^5^ In the MPACT trial, the combination of nab-paclitaxel plus gemcitabine also improved survival compared with gemcitabine alone (8.5 vs 6.7 months; HR, 0.72 95% CI, 0.62-0.83; *P* < .001).^6^ Most recently, the JCOG (Japan Clinical Oncology Group) 1611^7^ and NAPOLI 3^8^ trials showed conflicting results comparing triplet vs doublet chemotherapy regimens. In the NAPOLI 3 trial,^8^ the median overall survival with NALIRIFOX (fluorouracil, liposomal irinotecan, oxaliplatin, and leucovorin) was 11.1 (95% CI, 10.0-12.1) compared with 9.2 (95% CI, 8.3-10.6) months for nab-paclitaxel plus gemcitabine in first-line therapy (HR, 0.83; 95% CI, 0.70-0.99; *P* = .04). These results led to the US Food and Drug Administration (FDA) approval of NALIRIFOX as a treatment option for patients with metastatic PDAC.^9^ In practice, for clinically fit patients in Western countries, modified FOLFIRINOX has become the preferred first-line regimen for metastatic PDAC.

Activating mutations in the *KRAS* (OMIM 190070) oncogene are seen in up to 90% of patients with PDAC.^10^ Most *KRAS* mutations in PDAC are early events and involve codon 12. These are followed by additional alterations in tumor suppressor genes such as *CDKN2A* (OMIM 600160), *TP53* (OMIM 191170) or *SMAD4* (OMIM 600993) that contribute to accelerated progression of the disease.^11,12^

RAS proteins cycle between “on” and “off” conformations conferred by the binding of guanosine triphosphate (GTP) and guanosine diphosphate (GDP), respectively.^13^ The predominant mechanism of oncogenic activation depends on which codon is involved. For the most common G12 mutations, the predominant mechanism of oncogenic activation is via impairment of GTPase-activating protein hydrolysis. In G13, the predominant mechanism is an acceleration of GDP-to-GTP nucleotide exchange.^14^ Despite its high prevalence across different cancer types, *KRAS* was considered untreatable with drugs for decades. This was partly due to its smooth surface (only providing shallow pockets for drug binding) and picomolar affinity for GTP. The discovery of a switch II pocket prompted the development of molecules covalently bound to G12C and locking the protein in its GDP conformation.^15^ Two of these agents, sotorasib^16^ and adagrasib,^17^ have already attained accelerated approval by the FDA for the treatment of G12C-mutant non–small cell lung cancer (NSCLC), and a new wave of G12C inhibitors with perhaps improved activity are already in clinical trials.^18,19,20,21^ For the rare subset of patients with PDAC and *KRAS* G12C mutations (<1%), both sotorasib and adagrasib have shown modest improvement in survival, leading to their inclusion in National Comprehensive Cancer Network guidelines.^22,23^ However, while none of these G12 inhibitors have yet been approved by the FDA, their activity in PDAC is modest and likely to be improved if combined with epidermal growth factor receptor inhibitors.^24^

There is a lack of high-volume data exploring the outcomes of patients with metastatic PDAC treated with cytotoxic regimens based on the tumor-specific *KRAS* permutations. With increasing KRAS inhibitors under development, the need for large-scale, mutation-specific data are apparent. Using a clinical data approach from a nationwide deidentified electronic health record (EHR) database, we performed a genomic analysis of metastatic PDAC to identify the incidence of *KRAS* mutations and their association with clinical outcomes based on the most common treatments used in this disease.

## Methods

This study was approved by the Institutional Review Board at the University of Utah (a National Cancer Institute Comprehensive Cancer Center), which waived the requirement for informed consent due to the use of deidentified data. The study fully complied with the US patient confidentiality regulations, including adherence to the Health Insurance Portability and Accountability Act of 1996. The study followed the Strengthening the Reporting of Observational Studies in Epidemiology (STROBE) reporting guideline.

### Study Design

We used clinical data from the nationwide (US-based) Flatiron Health and Foundation Medicine Inc (FH-FMI) deidentified clinicogenomic database (CGDB). The deidentified data originated from approximately 280 US cancer clinics (approximately 800 sites of care). Retrospective longitudinal clinical data were derived from EHR data, comprising patient-level structured and unstructured data, curated via technology-enabled abstraction, and were linked to genomic data derived from FMI comprehensive genomic profiling (CGP) tests in the FH-FMI CGDB by deidentified, deterministic matching.^25^ Race and ethnicity were included as a social determinant of health and mapped from EHR data, where they were typically self-reported on patient intake forms. The FH-FMI–reported categories were Asian, Black or African American, Hispanic or Latino, White, and other (including American Indian and Alaska Native, Native Hawaiian or Other Pacific Islander, and multiracial). The data were deidentified and subject to obligations to prevent reidentification and protect patient confidentiality.

**Figure 1.**
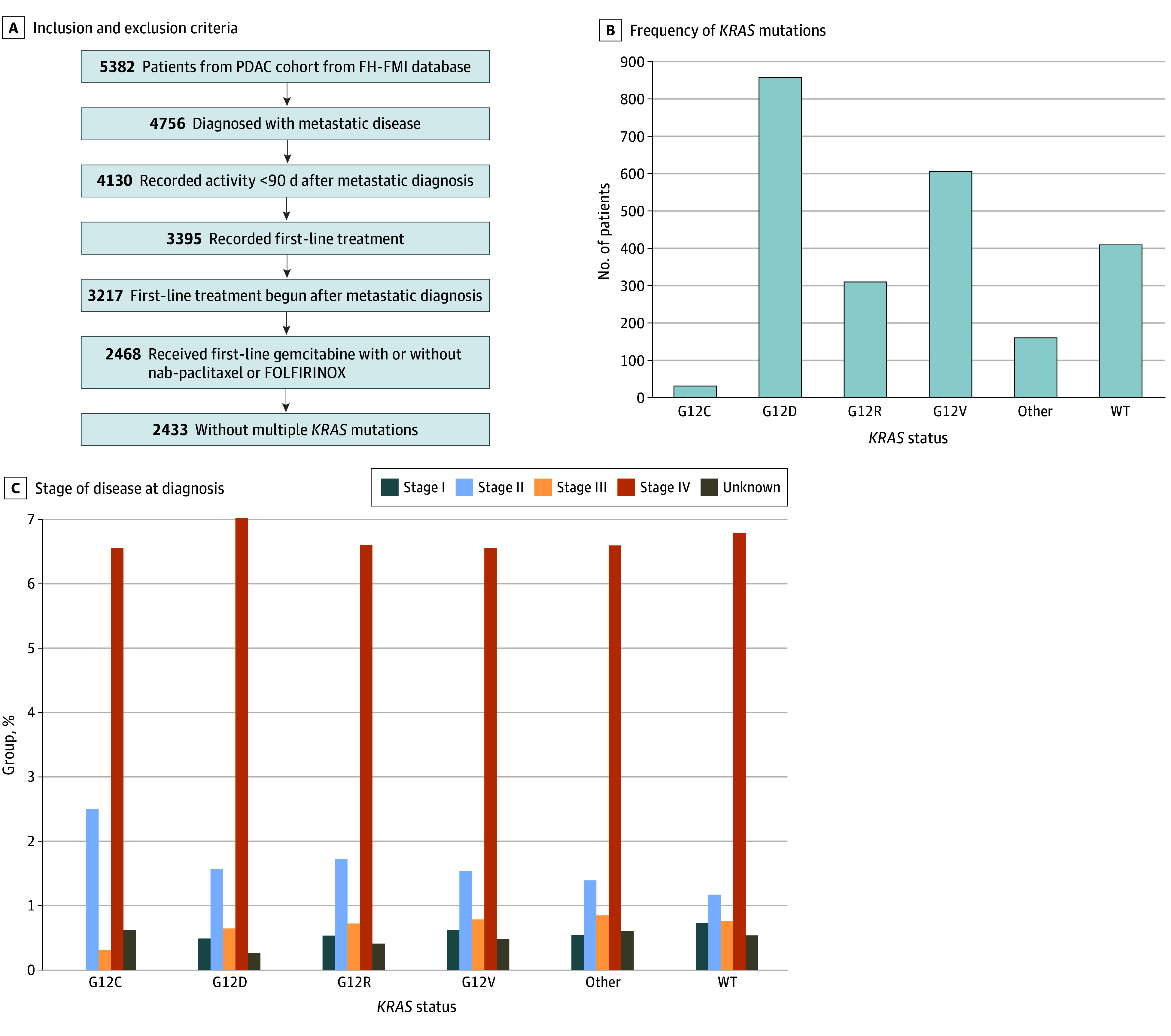
Initial Cohort Information FH-FMI indicates Flatiron Health and Foundation Medicine Inc; FOLFIRINOX, fluorouracil, irinotecan, oxaliplatin, and leucovorin; PDAC, pancreatic ductal adenocarcinoma; and WT, wild type.

We identified 5382 patients with pancreatic cancer within the FH-FMI CGDB. We included patients with metastatic disease with adequate follow-up time and available treatment data. Of the initial cohort, 2433 patients were selected for further analysis. A complete flowchart of the patient exclusion process is shown in Figure 1A.

Patients were categorized into 6 different *KRAS* mutation groups: wild type (WT), G12C, G12D, G12R, G12V, and other, including all other *KRAS* mutations (G12 and G13 and mutations at multiple codons in the *KRAS* gene). Based on the 3 most common chemotherapy regimens used, we categorized patients into first-line treatment groups, as receiving FOLFIRINOX, monotherapy, or gemcitabine with nab-paclitaxel.

Time to next treatment (TTNT) was defined as the recorded length for a given treatment line, including maintenance treatment, until the initiation of the next treatment line, the patient’s death, or last confirmed activity. Overall survival (OS) was defined as the time from first-line treatment initiation to death.

### Foundation Medicine CGP

Genomic alterations were identified via CGP obtained for routine clinical care of greater than 300 cancer-related genes on FMI’s next-generation sequencing test sequencing platforms (FoundationOne CDx and FoundationOne; FMI).^26^ For tissue biopsy samples, DNA was extracted from 40 μm of formalin-fixed paraffin-embedded sections, and CGP was performed on hybridization-captured, adapter ligation–based libraries to a mean coverage depth of greater than 500 times for 300 cancer-related genes and selected introns from 28, 31, or 36 genes frequently rearranged in cancer.^26^ For the comutation analysis, we included point mutations, truncations, rearrangements, and homozygous deletions.

### Statistical Analysis

Kaplan-Meier curves were generated using lifelines package, version 0.27.8 (Python Software Foundation).^27^ Patients were right-censored for first-line TTNT if they did not have a recorded second-line treatment and did not have a recorded death date. Patients were right censored for OS if they did not have a recorded death date. Median survival time and 95% CIs were also generated with the lifelines package.

All Cox proportional hazards models were generated using the lifelines package. All hazard models included possible confounding variables to account for patient differences between sample groups. These included the primary location of the tumor, age at first-line treatment, albumin at first-line treatment, sex, history of surgical resection, mutation of *RAF*, Eastern Cooperative Oncology Group performance status at first-line treatment, and race and ethnicity. Distributions of confounding variables across *KRAS* mutations and WT are in Table 1. Missing confounding variable values were imputed with median imputation (maximum missingness: Eastern Cooperative Oncology Group performance status of 11.6%). Statistical significance was set at 2-sided *P* < .05.

**Table 1.** Clinical Variables at Time of Treatment Initiation Across Patients With *KRAS* Mutations and *KRAS* WT Pancreatic Ductal Adenocarcinoma

Characteristic	Patient group, No. (%)		*P* value
	*KRAS* mutations (n = 2023)	*KRAS* WT (n = 410)	
Age at initiation of first-line therapy, mean (range), y	67.0 (66.59-67.41)	67.0 (66.0-68.0)	.62
Sex			
Female	916 (45.3)	177 (43.2)	.47
Male	1107 (54.7)	233 (56.8)	
Race and ethnicity			
Asian	37 (1.8)	13 (3.2)	.34
Black or African American	151 (7.5)	34 (8.3)	
Hispanic or Latino	5 (0.2)	0	
White	1365 (67.5)	273 (66.6)	
Other^a^	465 (23.0)	90 (22.0)	
First-line regimen			
FOLFIRINOX	923 (45.6)	172 (42.0)	.27
Gemcitabine	88 (4.3)	23 (5.6)	
Gemcitabine plus nab-paclitaxel	1012 (50.0)	215 (52.4)	
Stage at diagnosis			
I	108 (5.3)	30 (7.3)	.08
II	321 (15.9)	48 (11.7)	
III	144 (7.1)	31 (7.6)	
IV	1372 (67.8)	279 (68.0)	
Unknown or not documented	78 (3.9)	22 (5.4)	
Location of pancreatic primary tumor			
Body	386 (19.1)	70 (17.1)	<.001
Head	969 (47.9)	210 (51.2)	
Overlapping sites	212 (10.5)	44 (10.7)	
Tail	433 (21.4)	60 (14.6)	
NOS	23 (1.1)	26 (6.3)	
History of resection			
No surgery	1491 (73.7)	328 (80.0)	.009
Surgery	532 (26.3)	82 (20.0)	
ECOG performance status^b^			
0-1	1582 (78.2)	309 (75.4)	.59
2-3	211 (10.4)	47 (11.5)	
4	1 (0.05)	0	
Unknown	229 (11.3)	54 (13.2)	

Abbreviations: ECOG, Eastern Cooperative Oncology Group; FOLFIRINOX, fluorouracil, irinotecan, oxaliplatin, and leucovorin; NOS, not otherwise specified; WT, wild type.

^a^ Includes American Indian or Alaska Native, Native Hawaiian or Other Pacific Islander, and multiracial.

^b^ Range, 0 (normal activity) to 4 (completely bed- or chairbound); 5 indicates death and was not included in the study.

## Results

### Distribution of Patients 

The study included 2433 patients diagnosed with PDAC from February 9, 2010, to September 20, 2022, who underwent FMI CGP testing and had sufficient data available for the study end points (mean age at first treatment, 67.0 [range, 66.0-68.0] years; 1093 female [44.9%] and 1340 male [55.1%]) (Figure 1A). Fifty patients (2.1%) were Asian; 185 (7.6%), Black or African American; 5 (0.2%) Hispanic or Latino; 1638 (67.3%), White, and 555 (22.8%), other. As expected, most patients (2023 [83.1%] had tumors harboring a *KRAS* mutation). The age at first-line therapy and distribution by sex were similar in tumors with *KRAS* mutations and *KRAS* WT, with slightly higher male prevalence (1107 [54.7%] and 233 [56.8%], respectively). Racial and ethnic distribution was balanced across both groups (Table 1). The stage of disease at the time of diagnosis was predominantly de novo stage IV in tumors with *KRAS* mutations (1372 [67.8%]) and *KRAS* WT (279 [68.0%]). Most patients in either group had not undergone surgical resection (Table 1). The distribution of first-line regimens was similar, with 923 patients in the *KRAS* mutation group (45.6%) 172 in the *KRAS* WT group (42.0%) receiving FOLFIRINOX and 1012 (50.0%) and 215 (52.4%), respectively, receiving gemcitabine with nab-paclitaxel.

Among the 2023 tumors harboring a *KRAS* mutation, G12D was the most common (883 [43.6%]), followed by G12V (624 [30.8%]), G12R (319 [15.8%]), other (165 [8.2%]), and G12C (32 [1.6%]) (Figure 1B). The stage of disease on diagnosis was similar across all mutation groups (Figure 1C).

### Risk of Metastatic Disease and TTNT

**Figure 2.**
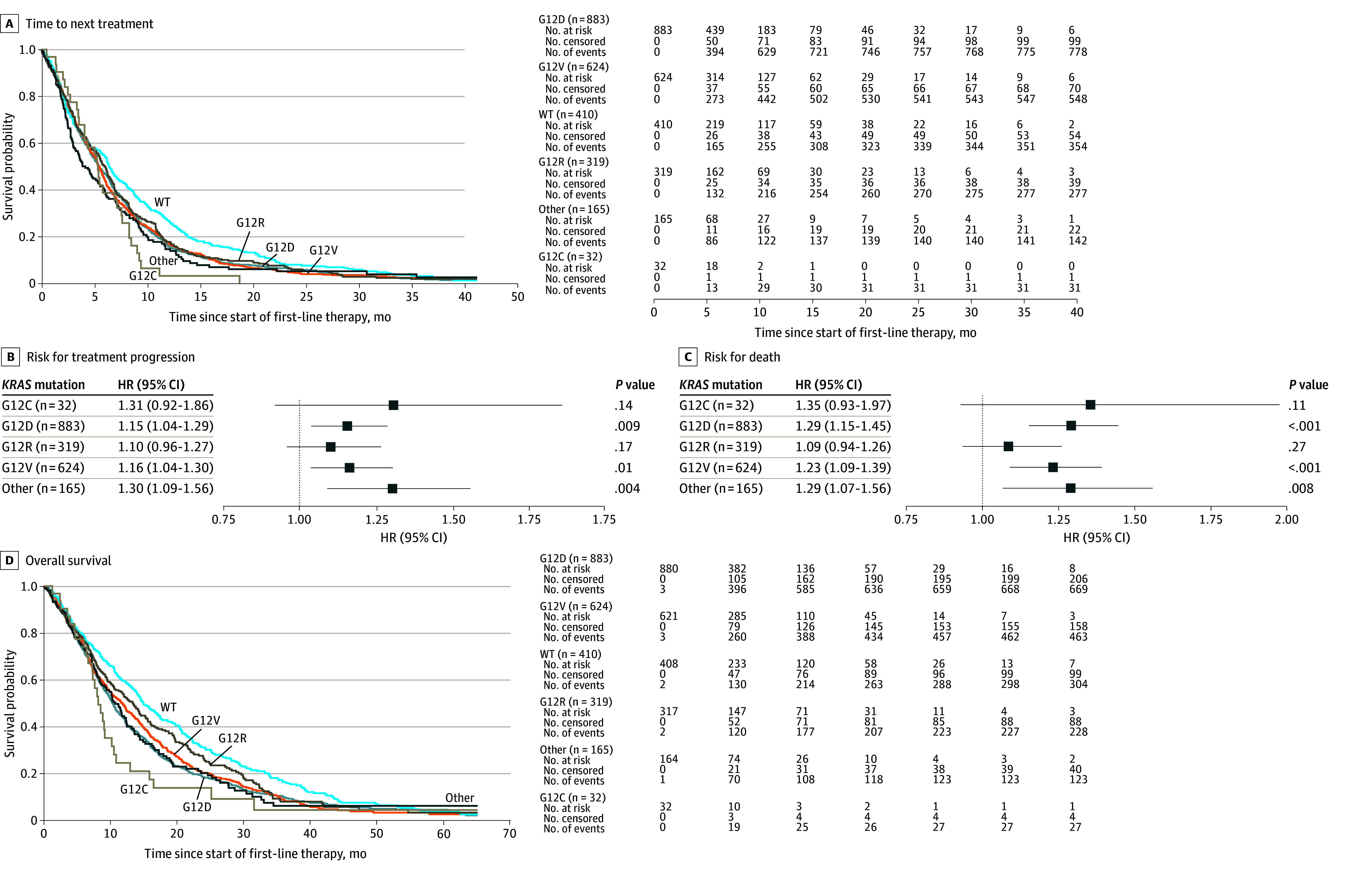
Risk for Treatment Progression and Death A, Kaplan-Meier curve of time-to-next-treatment across *KRAS* mutations (*P* = .008. multivariable log-rank analysis). A total of 280 patients were censored. B, Hazard ratios (HRs) from multivariate Cox proportional hazards model for treatment progression, with *KRAS* wild type (WT) as reference. C, Kaplan-Meier curve of overall survival across *KRAS* mutations (*P* < .001, multivariable log-rank analysis). A total of 595 patients were censored. D, HRs from multivariate Cox proportional hazards model for death, with *KRAS* WT as reference. Error bars indicate 95% CIs.

A χ^2^ analysis of stage at diagnosis found no significant difference between each *KRAS* mutation for risk of metastatic disease. Patients with metastatic PDAC with *KRAS* WT disease had longer median TTNT with first-line therapy (6.4 [95% CI, 5.8-7.1] months) than those with *KRAS* mutations (5.5 [95% CI, 5.2-5.7] months; *P* < .001). A multivariate log-rank analysis showed that TTNT was statistically different across different *KRAS* mutations. Numerically, *KRAS* G12C had the shortest median TTNT for first-line therapy at 5.3 (95% CI, 3.6-7.1) months, while G12R had the longest TTNT at 6.0 (95% CI, 5.2-6.6) months. Risk for disease progression was significantly higher for patients with G12V (HR, 1.16; 95% CI, 1.04-1.30; *P* = .01), G12D (HR, 1.15; 95% CI, 1.04-1.29; *P* = .009), and other (HR, 1.30; 95% CI, 1.09-1.56; *P* = .004) *KRAS* mutations compared with *KRAS* WT (Figure 2A and B).

### *KRAS* Mutations and OS

Patients with *KRAS* G12C tumors had the shortest median OS numerically at 8.0 (95% CI, 6.5-10.1) months from starting first-line treatment; however, the sample size was limited (n = 32). Patients with G12R mutations had the longest median OS at 13.2 (95% CI, 10.6-15.2) months. Overall, patients with *KRAS* mutations had a median OS of 10.7 (95% CI, 10.1-11.5) months, compared with 14.8 (95% CI, 13.7-17.1) months for *KRAS* WT (*P* < .001). Risk for death was significantly higher for patients with G12D (HR, 1.29; 95% CI, 1.15-1.45; *P* < .001), G12V (HR, 1.23; 95% CI, 1.09-1.39; *P* < .001), and other (HR, 1.29; 95% CI, 1.07-1.56; *P* = .008) *KRAS* mutations compared with *KRAS* WT (Figure 2C and D).

### First-Line Therapies and TTNT

First-line FOLFIRINOX treatment was associated with the longest median TTNT for tumors with *KRAS* mutation (5.8 [95% CI, 5.4-6.4] months) and *KRAS* WT (7.3 [95% CI, 6.2-9.2] months). FOLFIRINOX also led to the longest median TTNT across all common *KRAS* G12 mutations (Table 2). FOLFIRINOX was associated with a lower risk for treatment progression than gemcitabine plus nab-paclitaxel (HR, 1.19; 95% CI, 1.09-1.29; *P* < .001) and gemcitabine (HR, 1.37; 95% CI, 1.11-1.69; *P* = .003) (eFigure in Supplement 1).

**Table 2.** Median TTNT and OS Across First-Line Treatments and *KRAS* Mutations

*KRAS* Mutation	Treatment, median (95% CI), mo		
	FOLFIRINOX	Gemcitabine	Gemcitabine plus nab-paclitaxel
**Time to next treatment**			
G12C	5.2 (2.2 to 8.3)	NA	5.4 (3.6 to 7.6)
G12D	6.2 (5.3 to 6.8)	3.1 (1.5 to 5.0)	5.1 (4.5 to 5.8)
G12R	6.3 (5.2 to 8.1)	3.9 (1.6 to 6.2)	5.7 (4.7 to 6.7)
G12V	5.5 (4.7 to 6.4)	4.8 (2.4 to 8.4)	5.4 (4.8 to 5.9)
Other	4.4 (3.0 to 7.1)	2.8 (0.0 to 6.9)	4.6 (2.9 to 5.9)
All	5.8 (5.4 to 6.4)	3.8 (2.5 to 5.0)	5.3 (4.8 to 5.7)
WT	7.3 (6.2 to 9.2)	3.9 (1.9 to 8.5)	6.2 (5.2 to 6.9)
All	6.2 (5.6 to 6.5)	3.8 (3.0 to 4.8)	5.4 (5.1 to 5.8)
**Overall survival**			
G12C	8.9 (3.8 to 10.7)	NA	7.9 (4.6 to 10.4)
G12D	12.1 (10.4 to 13.3)	5.7 (2.6 to 9.3)	9.3 (8.2 to 10.2)
G12R	13.2 (10.6 to 15.5)	8.3 (2.4 to 12.1)	14.0 (9.9 to 16.5)
G12V	13.5 (11.4 to 15.0)	11.3 (6.0 to 19.4)	10.0 (8.4 to 12.1)
Other	12.2 (10.1 to 14.3)	6.3 (−0.2 to 15.1)	8.6 (7.0 to 12.3)
All	12.2 (11.5 to 13.4)	7.2 (5.7 to 9.5)	9.9 (9.1 to 10.5)
WT	18.5 (14.4 to 22.1)	10.3 (4.4 to 21.3)	13.9 (11.2 to 15.5)
All	13.2 (12.2 to 14.1)	8.0 (6.1 to 10.0)	10.4 (9.6 to 11.2)

Abbreviations: NA, not applicable; OS, overall survival; FOLFIRINOX, fluorouracil, irinotecan, oxaliplatin, and leucovorin; TTNT, time to next treatment; WT, wild type.

### First-Line Therapies and OS

FOLFIRINOX therapy was associated with the longest OS for all patients regardless of the tumor *KRAS* mutation status, with a median OS of 12.2 (95% CI, 11.5-13.4) months for *KRAS* mutations and 18.5 (95% CI, 14.4-22.1) months for *KRAS* WT. FOLFIRINOX also led to the longest median OS across all common *KRAS* G12 mutations, except for G12R (see Table 2). Overall, FOLFIRINOX was associated with a significantly lower risk for death compared with gemcitabine plus nab-paclitaxel (HR, 1.18; 95% CI, 1.07-1.29; *P* < .001) and gemcitabine (HR, 1.41; 95% CI, 1.13-1.75; *P* = .002) (eFigure in Supplement 1).

### Comutations

**Figure 3.**
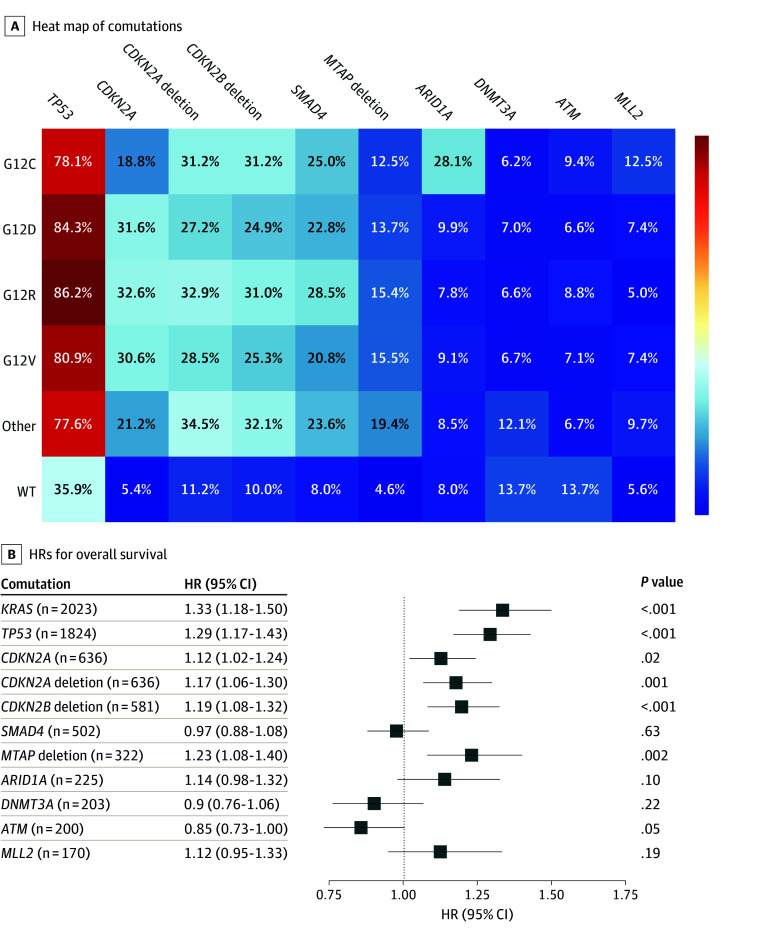
Comutations with *KRAS* A, Heat map of the 10 most common comutations among cohort, across *KRAS* mutations. Bar scale ranges from 4% (blue) to 87% (red). B, Hazard ratios from multivariate Cox proportional hazards model of *KRAS* mutations and 10 most common comutations, with wild type of each gene as reference.

In addition to *KRAS,* the 5 assayed genes with the highest mutation rates across all patients were *TP53, CDKN2A* (homozygous deletion), *CDKN2A, CDKN2B* (homozygous deletion), and *SMAD4.* A multivariate Cox proportional hazards regression analysis showed that the mutant state of the 5 most common comutations had a significantly higher risk of death compared with *KRAS* WT, except for *SMAD4* (Figure 3). *TP53* (86.2%; *P* = .10), *CDKN2A* (32.6%; *P* = .39), and *SMAD4* (28.5%; *P* = .02) each had the highest comutation rates with *KRAS* G12R compared with other *KRAS* mutations (Figure 3A). There were no significantly different comutation rates for *CDKN2A* deletion (range, 27.2%-34.5%; *P* = .12) or *CDKN2B* deletion (range, 24.9%-32.1%; *P* = .07) across *KRAS* mutations. Among the top 10 genes with the highest mutation rates across all patients, the *MTAP* deletion ranked sixth and was associated with a significantly higher risk of death compared with *KRAS *WT (HR,1.23; 95% CI, 1.08-1.40; *P* = .002) (Figure 3B). The incidence of additional genomic alterations across all patients affecting pathways with therapeutic potential were as follows: 229 patients (9.4%) had mutations in *BRCA1*, *BRCA2*, *PALB2*, or *RAD51*; 154 (6.3%) in *PIK3CA*, *AKT1*, *AKT2*, or *MTOR*; 54 (2.2%) in *PDGFRA* or *PDGFRB*; and 10 (0.4%) in *VEGFA*.

## Discussion

In this cohort study, we used clinical data to assess the association of *KRAS* mutations in metastatic PDAC with the clinical outcomes and responses to first-line chemotherapy regimens. We included 2433 patients for analysis whose tumors had undergone genomic sequencing and had adequate clinical data available. This was a multi-institutional study across different care settings, academic status, and community practices.

Consistent with prior literature, most tumors harbored a *KRAS* mutation (2023 [83.1%]). Overall, patients with *KRAS* WT disease had longer TTNT during first-line therapy and OS (6.4 and 14.8 months, respectively) compared with *KRAS* mutations (5.5 and 10.7 months, respectively). Among the tumors with *KRAS* mutations, those with G12C had the shortest TTNT among the single G12 mutation groups (5.3 months) and the shortest OS (8.0 months). However, these results should be interpreted cautiously as they did not reach statistical significance, likely due to the low number of patients in this group (n = 32). Keane et al^28^ also recently reported no difference in clinical outcomes in patients with PDAC and *KRAS* G12C (39 of 3671) vs non-G12C mutations across all stages. G12D, G12V, and other *KRAS* mutations were associated with the highest risk of death compared with *KRAS* WT disease.

First-line FOLFIRINOX therapy was associated with numerically improved TTNT and OS compared with gemcitabine with or without nab-paclitaxel across *KRAS* G12D and G12V, the most common G12 mutations. This was reflected in both median times and results of multivariate hazard analyses. Our multivariate hazard analysis controlled for common mutations, prognostic biomarkers, and patient demographic characteristics, adding to the evidence that a triplet combination of cytotoxic treatment in Western patients is associated with improved outcomes.^8,29^

The present study is important due to the evolving treatment landscape of metastatic PDAC, which is poised to change with the emergence of RAS inhibitors. It provides benchmark survival data for patients with metastatic PDAC and RAS mutations. These data will inform end points of upcoming clinical trials testing RAS inhibitors and chemotherapy combinations. Direct inhibition of the RAS family proteins has only been possible recently after decades of research efforts. Recently, the FDA approved the first allele-specific KRAS inhibitors (sotorasib and adagrasib) targeting KRAS G12C inNSCLC.^16,17^ Both drugs have modest efficacy as monotherapy in PDAC with a *KRAS* G12C mutation.^22,23^ Nevertheless, most PDAC is driven by *KRAS* G12D, G12V, or G12R mutations, as shown in the current study and others.^30,31^ Numerous KRAS inhibitors are currently in preclinical development and early clinical trials.^32^

One inhibitor for which preliminary clinical data are available is RMC-6236, a pan-RAS inhibitor that targets GTP-bound RAS proteins. Early efficacy data from an ongoing first-in-human study was recently reported.^33^ Sixty-five patients with heavily pretreated metastatic PDAC were included, and 46 had evaluable disease with a response rate of 20% and, more importantly, a disease control rate of 87%.^34^ These preliminary results indicate activity in PDAC with *KRAS* G12 mutations (G12C excluded), with further confirmation pending upon the final trial results. In addition, RMC-6236 is also active in patients whose tumors harbored Q61X mutations (PDAC and melanoma) and V600E *BRAF*–mutated colorectal cancer with emergent RAS mutations following treatment with BRAF and epidermal growth factor receptor inhibitors.^35^ Considering the highest risk of death of patients with PDAC and G12D, G12V, and other *KRAS* mutations, if the activity of such inhibitors is confirmed, the outcomes of these patients may improve.

Among the tumors with *KRAS* G12 mutations in our cohort, it is noteworthy that *KRAS* G12R was present in 319 patients (13.1%) but was not associated with increased risk of progression to first-line therapy or death, unlike *KRAS* G12D and G12V. These findings are consistent with preclinical studies in organoid models showing that *KRAS* G12R mutations are distinct, induce a less pronounced *KRAS* transcriptional signature, are less likely to induce premalignant pancreatic intraepithelial neoplasia lesions, and perhaps follow a different progression model with more favorable biology.^24^ This hypothesis is also supported by recent clinical data. In an analysis of 5550 patients with PDAC,^30^ patients with *KRAS* G12R mutations had significantly longer OS compared with those with G12D mutations (396 vs 311 days; HR, 0.81; 95% CI, 0.74-0.88; *P* < .001). In a single-institution study with 703 patients with PDAC of all stages,^31^ those with *KRAS* G12R mutations had similar OS (median, 34 months; HR, 1.00; 95% CI, 0.71-1.50; *P* = .88) compared with *KRAS* WT disease (median, 38 months).^31^ In addition, there was a higher prevalence of *KRAS* G12R mutations in well and moderately differentiated tumors than in poorly differentiated or anaplastic tumors (14% vs 9%; odds ratio,1.70; 95% CI, 1.05-2.99; *P* = .04). In the same study,^31^ patients with metastatic PDAC with *KRAS* G12D mutations had a shorter median OS (11 months) compared with those with *KRAS* G12R mutations and *KRAS* WT tumors (median OS, 25 and 24 months, respectively). In a smaller single-institution cohort of 264 patients with metastatic PDAC and *KRAS* mutations,^36^ G12R was present in 12% of the tumors (n = 32), and there were no differences in the median OS across the different G12 mutations, likely due to overall low sample size.

In our dataset, *KRAS* G12R tumors had the highest incidence of comutations with the suppressor genes *SMAD4*, *TP53*, and *CDKN2A*, but this difference was only significant for *SMAD4*. It is plausible that the G12R mutation has less oncogenic potency than other common G12 mutations and can explain the overall rarity of this mutation in cancer in general.^11^ *KRAS* G12R exhibits unique properties compared with other common G12 *KRAS* mutations. Phosphatidylinositol 3-kinase (PI3K) is one of the main effector RAS pathways, and the RAS-PI3K interaction is vital in RAS-driven tumorigenesis, tumor maintenance, and metastasis.^37^ Due to structural perturbation, G12R upregulates KRAS-independent PI3K activity directly, unlike the more common G12, G12D, or G12V variants in PDAC.^38^ The defective PI3Kα signaling in G12R compared with other G12 mutations in PDAC may impact the drivers of resistance to KRAS inhibitors and may ultimately require different therapeutic approaches.

### Limitations

The limitations of this study pertain to the heterogeneity of clinical data. Data were collected retrospectively and are subject to residual confounding. In addition, the sample size was limited for certain mutational groups, particularly *KRAS* G12C, leading to limited power for statistical analyses of that group. Another potential limitation is the detection rate of genomic alterations in general by the commercially available assays, which is particularly pertinent in PDAC due to its high stromal content and low cellularity.

## Conclusions

In this cohort study of 2433 patients, we report the differential association of *KRAS* mutations on clinical outcomes in patients with metastatic PDAC. This large, multi-institutional cohort of patients with PDAC offers detailed insights into outcomes of allele-specific *KRAS* mutations with specific treatments. We found that certain *KRAS* permutations were associated with worse outcomes with cytotoxic chemotherapy regimens and a higher risk of death. We also found that a triplet chemotherapy regimen (FOLFIRINOX) in the first-line setting was associated with a better TTNT and OS across most patient groups. In addition, *KRAS* G12R was associated with more favorable clinical outcomes and had the highest incidence of comutations with the most affected tumor suppressor genes in PDAC. This supports the notion of lower oncogenic activity of this variant. In its totality, these data set a benchmark for future studies on KRAS inhibitors for specific *KRAS* variants and highlights the groups for which treatment combinations may ultimately be necessary.

## Notes

Supplement 1. **eFigure.** Time to Next Treatment and Overall Survival Across Common First-Line Treatments

Supplement 2.
Data Sharing Statement
