# Supplementary material for: KRAS Mutation Status and Treatment Outcomes in Patients With Metastatic Pancreatic Adenocarcinoma
Source: JAMA Netw Open. 2025 Jan 7;8(1):e2453588. doi: 10.1001/jamanetworkopen.2024.53588 (PMC11707629; doi:10.1001/jamanetworkopen.2024.53588)
Supplement: Supplement 1. — eFigure. Time to Next Treatment and Overall Survival Across Common First-Line Treatments [file jamanetwopen-e2453588-s001.pdf]

Supplementary Online Content

Norton C, Shaw MS, Rubnitz Z, et al. *KRAS* mutation status and treatment outcomes in patients with metastatic pancreatic adenocarcinoma. *JAMA Netw Open*. 2025;8(1):e2453588. doi:10.1001/jamanetworkopen.2024.53588

eFigure. Time to Next Treatment and Overall Survival Across Common First-Line Treatments

This supplementary material has been provided by the authors to give readers additional information about their work.

© 2025 Norton C et al. *JAMA Network Open*.

eFigure. Time to Next Treatment and Overall Survival Across Common First-Line Treatments

eFigure, A. Kaplan-Meier curve of time-to-next-treatment across first line treatment, with p-value from multivariable log-rank analysis

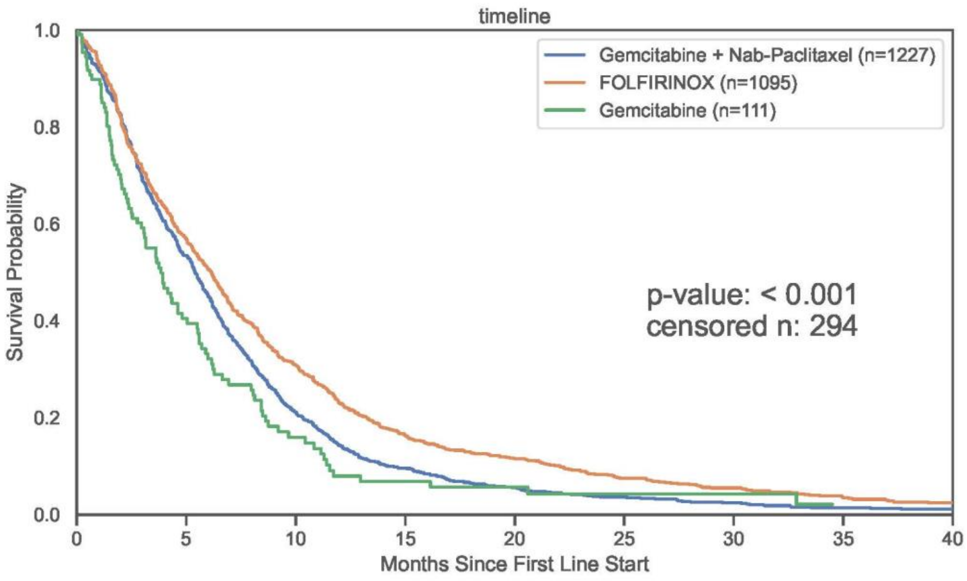

	0	5	10	15	20	25	30	35	40
**Gemcitabine + Nab-Paclitaxel (n=1227)**									
At risk	1227	597	221	90	45	28	17	10	6
Censored	0	83	109	123	132	133	136	136	138
Events	0	547	897	1014	1050	1066	1074	1081	1083
**FOLFIRINOX (n=1095)**									
At risk	1095	584	290	144	94	58	37	21	12
Censored	0	55	92	104	114	118	124	127	130
Events	0	456	713	847	887	919	934	947	953
**Gemcitabine (n=111)**									
At risk	111	39	14	6	4	3	3	0	0
Censored	0	12	14	14	15	15	15	17	17
Events	0	60	83	91	92	93	93	94	94

© 2025 Norton C et al. *JAMA Network Open*.

eFigure, B. Hazard ratios from multivariate Cox Proportional Hazard model for treatment progression, with FOLFIRINOX as reference

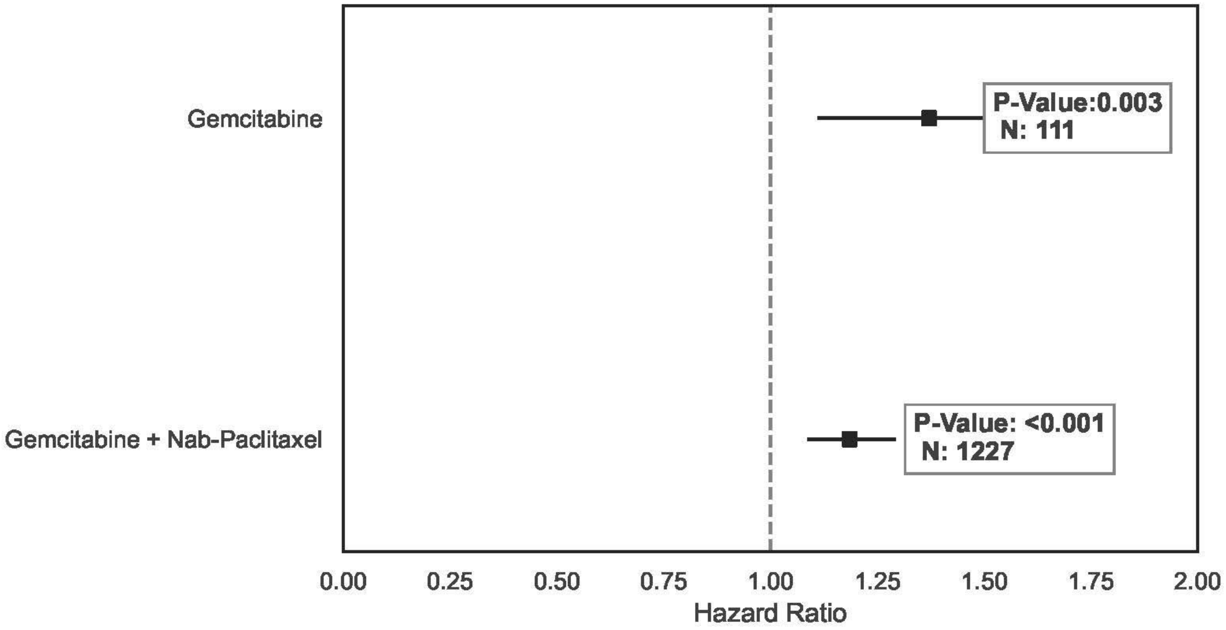

© 2025 Norton C et al. *JAMA Network Open*.

eFigure, C. Kaplan-Meier curve of overall survival across first line treatment, with p-value from multivariable log-rank analysis.

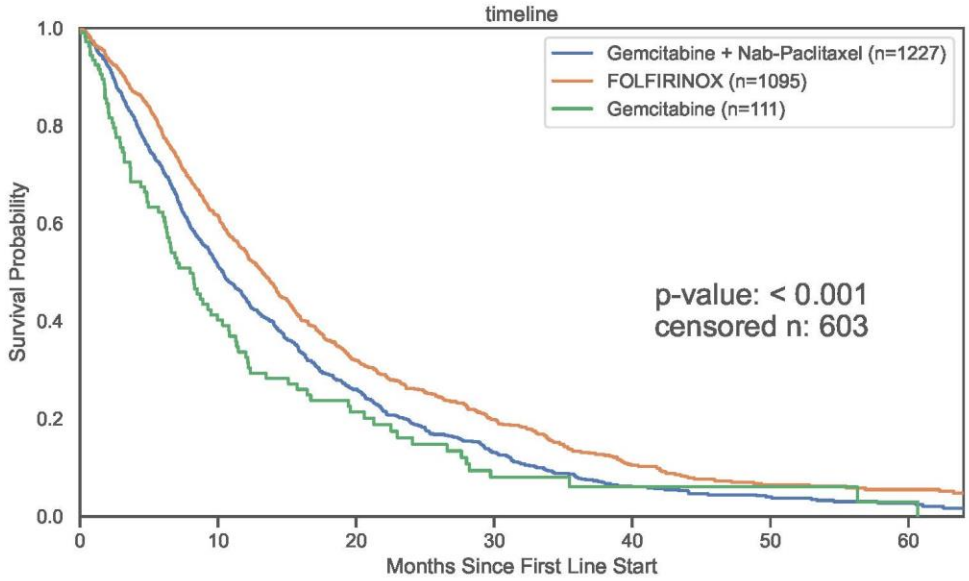

	0	10	20	30	40	50	60
**Gemcitabine + Nab-Paclitaxel (n=1227)**							
At risk	1221	528	209	81	32	18	8
Censored	0	151	235	268	275	279	284
Events	6	548	783	878	920	930	935
**FOLFIRINOX (n=1095)**							
At risk	1091	566	239	116	50	24	15
Censored	0	141	218	259	275	283	289
Events	4	388	638	720	770	788	791
**Gemcitabine (n=111)**							
At risk	110	37	18	6	3	2	1
Censored	0	15	17	19	21	22	22
Events	1	59	76	86	87	87	88

© 2025 Norton C et al. *JAMA Network Open*.

eFigure, D. Hazard ratios from a multivariate Cox Proportional Hazard model for death, with FOLFIRINOX as reference.

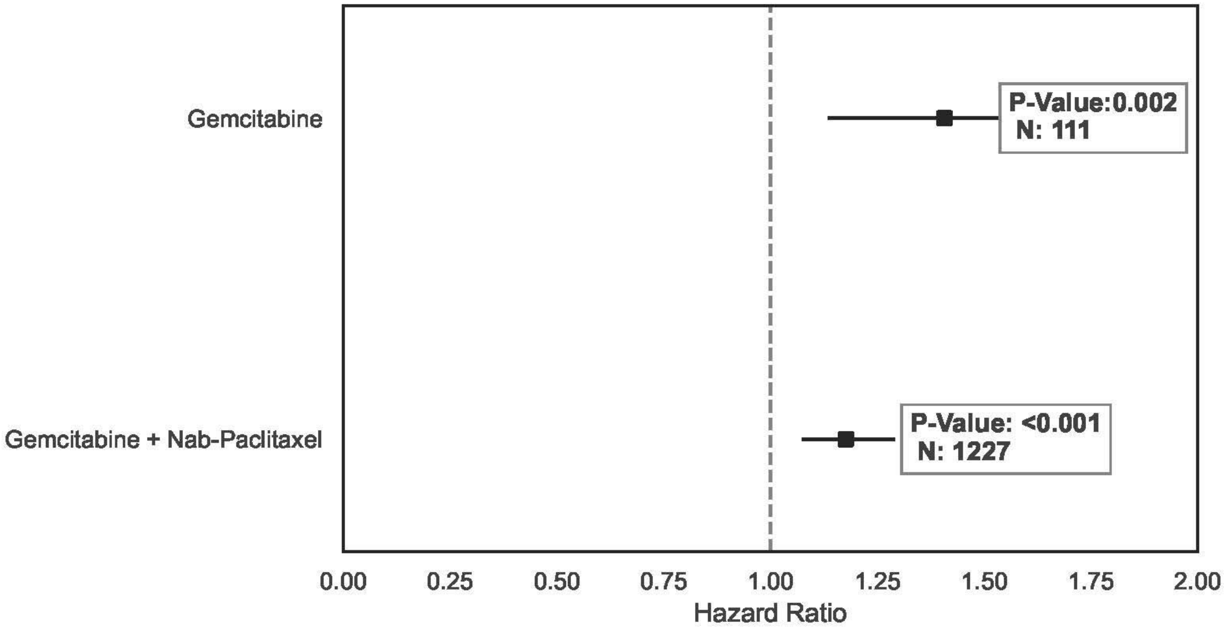

© 2025 Norton C et al. *JAMA Network Open*.
