# Supplementary material for: KRAS Mutation Status and Treatment Outcomes in Patients With Metastatic Pancreatic Adenocarcinoma
Source: JAMA Netw Open. 2025 Jan 7;8(1):e2453588. doi: 10.1001/jamanetworkopen.2024.53588 (PMC11707629; doi:10.1001/jamanetworkopen.2024.53588)
Supplement: Supplement 2. — Data Sharing Statement [file jamanetwopen-e2453588-s002.pdf]

Data Sharing Statement

Norton. KRAS Mutation Status and Treatment Outcomes in Patients With Metastatic Pancreatic Adenocarcinoma. *JAMA Netw Open*. Published January 07, 2025. doi:10.1001/jamanetworkopen.2024.53588

Data
Data available: Yes
Data types: Deidentified participant data
How to access data: The research data supporting this study was provided by Flatiron Health, Inc. This deidentified data set can be accessed upon request and is governed by a licensing agreement with Flatiron Health. Researchers interested in using these data should reach out to DataAccess@flatiron.com to discuss the terms of use.
When available: With publication

Supporting Documents
Document types: None

Additional Information
Who can access the data: Researchers whose proposed use of the data has been approved
Types of analyses: For a specified purpose.
Mechanisms of data availability: Researchers interested in using these data should reach out to DataAccess@flatiron.com to discuss the terms of use.
